# Spontaneously Fermented Fruiting Bodies of *Agaricus bisporus* as a Valuable Source of New Isolates of Lactic Acid Bacteria with Functional Potential

**DOI:** 10.3390/foods9111631

**Published:** 2020-11-08

**Authors:** Katarzyna Skrzypczak, Klaudia Gustaw, Ewa Jabłońska-Ryś, Aneta Sławińska, Waldemar Gustaw, Stanisław Winiarczyk

**Affiliations:** 1 Department of Plant Food Technology and Gastronomy, Faculty of Food Science and Biotechnology, University of Life Sciences in Lublin, 8 Skromna Street, 20-704 Lublin, Poland; ewa.jablonska-rys@up.lublin.pl (E.J.-R.); aneta.slawinska@up.lublin.pl (A.S.); waldemar.gustaw@up.lublin.pl (W.G.); 2 Department of Biotechnology, Microbiology and Human Nutrition, Faculty of Food Science and Biotechnology, University of Life Sciences in Lublin, 8 Skromna Street, 20-704 Lublin, Poland; klaudia.gustaw@up.lublin.pl; 3 Department of Epizootiology and Clinic of Infectious Diseases, University of Life Sciences in Lublin, Głęboka 30, 20-612 Lublin, Poland; genp53@interia.pl

**Keywords:** fermentation, *Agaricus bisporus*, lactic acid bacteria (LAB), MALDI-TOF Biotyper

## Abstract

The aim of the investigation was the identification and initial study of lactic acid bacteria (LAB) isolated from spontaneously fermented (at 28 °C for 5 days) fruiting bodies of white button mushrooms (*Agaricus bisporus*). The isolated LAB were preliminarily characterized applying the MALDI-TOF Biotyper. Moreover, further phenotypical, genotypical characteristics as well as some functional and technological properties of the selected microorganisms (including the ability to produce exopolysaccharides, cell hydrophobicity, resistance to low pH, and bile salt) were also analyzed. Among autochthonous LAB (isolated from the tested mushroom raw material), *Leuconostoc mesenteroides* predominated in spontaneously fermented *A. bisporus*, while *Lactiplantibacillus paraplantarum, Lactiplantibacillus plantarum*, and *Lactococcus lactis* were less abundant. The highest dynamics of acidification of the mushroom material were exhibited by isolates EK55 and EK4 that, after 24 h of incubation, were able to decrease the pH of the raw material up to 5.06 ± 0.021 and 5.17 ± 0.015, respectively. Furthermore, the analysis of bacterial cell hydrophobicity indicated that the highest values of this parameter were noted for *L. plantarum* isolates EK12 (29.59 ± 0.7%), EK55 (28.75 ± 0.551%), and EK5 (27.33 ± 1.516%). It was revealed some of the analyzed LAB (especially isolates *L. plantarum* EK55 and *L. paraplantarum* EK4) exhibited functional and technological potential that might be used in the formulation of novel starter cultures.

## 1. Introduction

The primary application of lactic acid bacteria (LAB) is related to preservation of food, but increasing relevance and contribution of these microorganisms in diverse sectors of food and pharmacy industries are observed. Moreover, this group of bacteria is an intrinsic factor in the production of various foodstuffs obtained through spontaneous fermentation.

Nowadays, LAB are widely used in food technology as commercial starter cultures. However, there is a need in the food industry to improve the properties of the starters used so far, whereas the development of novel fermented products (including new generation foodstuffs) often requires seeking new sources of microorganisms exhibiting desired technological and functional characteristics.

Extensive investigations have confirmed that food-derived LAB (in particular *Lactiplantibacillus* bacteria) exhibiting the ability to colonize the human intestine might play a significant role in its proper functioning. Additionally, there is a growing need to select new strains of bacteria with functional properties in order to improve food quality and broaden the range of fermented products, including probiotic ones [1]. Nevertheless, all microorganisms (especially new isolates) with the desired technological and functional potential have to be suitably tested to ensure the safety of application thereof in food industry. According to the guidelines recommended by FAO/WHO, any strains of bacteria that can potentially be used as food starter cultures and considered potentially probiotic must be thoroughly investigated. The initial phase of studies includes precise identification of the strain with phenotypic and genotypic methods. Modern molecular biology methods must be used to identify the genus, species, and strain of the microorganism in order to deposit it in the national culture collection [2].

Currently, new bacterial strains representing LAB are isolated and subjected to multidisciplinary investigations due to the constant need for finding useful microorganisms with valuable biotechnological, medical, and pharmaceutical potential and technological suitability for functional food production. This contributes to discovering new ecological niches, which are the sources of such bacteria with desired properties, e.g., health-promoting effects.

It is worth emphasizing that the fermentation process, based on natural microbiota consisting of autochthonous bacteria present in the environment and raw materials, often yields more satisfactory results than commercially available allochthonous starters [3]. Regrettably, most of the industrially available and widely used strains used for the fermentation of various plant-derived raw materials are allochthonous, and this type of microflora is not competitive with endogenous microbiota. In this regard, isolation and screening of LAB from spontaneously fermented foodstuff seem to be an interesting approach and a powerful means to obtain indigenous cultures useful for commercial applications [4]. 

Nowadays, mushrooms are recognized for their organoleptic characteristics as well as for their numerous bioactive substances which are beneficial and health-promoting [5]. Consumption of mushrooms has been found to exert a positive effect on fighting high blood pressure, high cholesterol, and even cancer [6,7]. Moreover, most edible mushrooms are perceived as a source of functional compounds, which, in combination with their low levels of fats and energy, makes them a great ingredient of a balanced diet [8]. It is also worth mentioning that in the dry mass of fruiting bodies of mushrooms, the content of bioavailable proteins is higher than that in most vegetables and fruits [5]. Nonetheless, relatively high protein concentration together with high enzymatic activity and low content of dry matter are the main factors influencing the low durability of mushroom fruiting bodies. However, some results suggest that LAB inoculates may be perceive as a proper strategy for improving the storage stability of spent mushroom (*Pleurotus ostreatus*) [9,10]. Hence, lacto-fermentation might constitute a desirable, biological method of preserving *A. bisporus* as an alternative to sterilization, canning, or pickling [9,10].

At present, the process of lactic acid fermentation as a biological method for conservation of fungal fruiting bodies is not commonly used on an industrial scale, although this was a very popular way of extending the durability of mushroom raw material in the middle of the last century. This relatively cost- and time-efficient preservation method may be regarded as sustainable management of excess raw material. Moreover, such a bio-conservation process yields a product with more desirable characteristics than those in salted food, which is mainly associated with a reduction in nutrient losses and a possibility to lower the salt concentration in brine [5]. 

Unfortunately, there are not many research studies referring the lactic fermentation of the fruiting bodies of edible mushrooms fruits [5]. Even though *A. bisporus* is the most commonly consumed in the world, to our knowledge, the spontaneous fermentation process of this fungal raw material as well as the associated microflora have not been well characterized. 

Therefore, the aim of the investigation was the detecting and initial identification of lactic acid bacteria (LAB) isolated from spontaneously fermented fruiting bodies of white button mushrooms (*A. bisporus*) and further genetical analyses as well as selected functional properties of the chosen bacteria. Moreover, the preliminary study was also focused on the detection of isolates of LAB demonstrating the most favorable properties and the highest potential for application in lactic fermentation of *A. bisporus*, as this may contribute to further development of a new range of functional fermented products derived from fungal raw material.

## 2. Materials and Methods 

### 2.1. Spontaneous Fermentation of Fungal Raw Material

The white button mushrooms (*A. bisporus*) used in the process of spontaneous fermentation were purchased from the company “*Growing mushrooms*-*Pawelec Zdzisław*” (Kazimierzówka 34, Świdnik, Poland). The mushrooms were collected in the morning, and the fruiting bodies were delivered to the laboratory on the same day (the research analysis began 3 h after harvesting). The mushrooms were washed under running (lukewarm) tap water, shredded into 2 mm thick slices, and flooded with a saline solution containing 2% (*w/v*) NaCl. After thorough mixing, the material was left until brine was released from the fruiting bodies through the exo-osmosis process. Then, the shredded mushrooms were divided into equal weight samples, which were transferred into sterile glass jars with a volume of 300 mL (three identical batches of fermentation material were prepared). Subsequently, the jars (containing mushrooms completely immersed in the brine) were tightly closed with lids and placed in a thermostatic chamber, where spontaneous fermentation was conducted (28 °C for 5 days).

### 2.2. Isolation and Selection of LAB Isolates

The procedure of bacteria isolation was performed according to [11] with some modifications. Briefly, 10 g of samples (containing the mushroom fruiting bodies with the brine) were collected from the fermented matrix and shaken for 5 min (Multi Speed Vortex MSV-3500, Biosan, Finland). Then, 1 mL of the liquid suspension was taken in order to prepare a series of consecutive dilutions in physiological saline. Thereafter, 100 μL of the respective dilutions were collected, transferred to Petri dishes with previously prepared MRS agar (BioMaxima, Lublin, Poland), spread thoroughly on the medium surface, and incubated anaerobically at 37 °C for 48 h (in duplicates). After the incubation, individual colonies of microorganisms (different from each other) were collected and again transferred to sterile de Man, Rogosa and Sharpe (MRS) agar (to ensure purity of the isolates, this stage of the procedure was performed twice). 

Isolates capable of lactic acid production were detected with the plate method [11]. For this purpose, 1% CaCO_3_ was added to MRS agar and, after inoculation, the tested isolates were incubated in anaerobic conditions (24 h/37 °C). Acid-producing bacterial colonies (with a clean surrounding zone) were randomly selected from MRS plates and purified by replication on MRS agar plates.

The purified isolates (exhibiting acidification abilities) were maintained at −80 °C in de Man, Rogosa and Sharpe (MRS) broth (Merck KGaA, Darmstadt, Germany) supplemented with 20% glycerol as stock cultures (to store tested collection of isolates). Prior to the analysis, each of the tested isolates was regularly transferred into fresh sterile broth MRS and incubated (37 °C/18 h) in anaerobic conditions [12].

Microscopic observations, catalase tests, and Gram staining were performed for initial screening of *Lactiplantibacillus* [13,14]. Individual colonies of microorganisms were identified phenotypically by Gram staining. The catalase test was performed with the use of 3% hydrogen peroxide solution that was dripped on single bacterial colonies (of each isolate) grown on MRS agar plates (no gas bubbles indicated catalase-negative microorganisms). Gram-positive, catalase-negative, bacilliform, and cocciform (spherical shape, bead-like chains) isolates were selected for further analysis.

### 2.3. Preliminary Identification of Isolates Using the MALDI-TOF Biotyper

One hundred and ten isolates of LAB bacteria were subjected to identification using the MALDI-TOF Biotyper (Bruker Daltonics, Bremen, Germany). The identification was performed according to the method described by [15]. In brief, after 24 h of incubation, a single bacterial colony (of each isolate) was suspended in 150 μL of sterile deionized water. In order to obtain homogenous mixtures, the suspensions were thoroughly mixed by vortexing. Thereafter, 96% ethanol (450 μL) was added to each sample, which was further vortexed for 1 min and centrifuged (8000× *g*/2 min/4 °C). After removing the supernatant, the step was repeated twice. Then, 40 μL of a formic acid solution (70%) were added to each sample, which was further vortexed. Subsequently, 40 μL of 99% acetonitrile were added, and this was followed by vortexing (1 min) and centrifugation (8000× *g*/2 min/4 °C). Samples for analysis (1 μL for each isolate in three repetitions) were placed on a metal plate and left to dry in a sterile chamber at room temperature. Then, 1 μL of HCCA-α-Cyano-4-hydroxycinnamic acid (10 mg/mL) was applied on each spot of the sample and again left to dry. After the incubation (at ambient temperature), the plate was subjected to analysis in an UltrafleXtreme MALDI-TOF mass spectrometer (Bruker, Germany) with a 1000 Hz neodymium-doped yttrium aluminum garnet nitrogen laser (Nd-YAG). The determination was performed with the use of the MALDI Biotyper 3.0 software package (Bruker, Germany), where a score on a scale from 0 to 3.0 exhibited the level of identification probability. 

A result above 2.0 implied reliable (unambiguous) identification of the genus and probable species identification.

In the study, *L. plantarum* DSM 20,174 was used as the reference strain (Leibniz Institut DSMZ-Deutsche Sammlung von Mikroorganismen und Zellkulturen GmbH, Germany).

### 2.4. Comparison of the Selected Technological Functional Properties of Selected Autochthonous LAB

#### 2.4.1. Ability of Isolates to Grow on Medium with an Alternative Carbon Source

The composition of MRS agar was modified by replacing glucose with freeze-dried mushrooms (2 g/L). The profile of saccharides in the used raw material has been further analyzed [16].

The sterile modified medium was inoculated by spreading 1% (vol/wt) of the tested isolate cell suspension (prepared in sterile saline from culture in the exponential growth phase and adjusted to final OD_600_ = 0.5) on its surface. The inoculated Petri dishes (in triplicate for each analyzed LAB isolate) were incubated for 48 h at 37 °C (in anaerobic conditions). Isolates that were able to form more than 30 colonies on the modified culture medium were considered capable of growth on an alternative carbon source (mushroom sugars). Isolates exhibiting such characteristics were marked with “+” and selected for the next stage of the investigations.

#### 2.4.2. Dynamics of Acidification of the Mushroom Material

In order to determine the dynamics of the pH decrease in the mushroom material, fresh fruiting bodies of white button mushrooms were homogenized using a hand-blender to obtain homogeneous mass (smooth matrix). Then, samples containing the mushroom material were sterilized (by autoclaving) and inoculated by 1% (vol/wt) of a bacterial cell suspension of a given LAB isolate (prepared in sterile saline from cultures in the exponential growth phase and adjusted to final OD_600_ = 0.5). The inoculated samples (in triplicate for each bacteria isolate) were incubated at 37 °C (in anaerobic conditions). The pH value was measured after 3, 7, and 24 h of fermentation. Non-inoculated samples were a control variant.

#### 2.4.3. Detection of Isolates Able to Produce Exopolysaccharides

The ability of the LAB isolates to synthesize exopolysaccharides (EPSs) was estimated with the method described by Manini et al. [17] with some modification. Briefly, the tested isolates were incubated on MRS agar plates for 48 h days at 37 °C. Triplicate plates containing 30 to 250 colonies (of each of the tested isolate) were evaluated for the ability to produce mucus. Colonies were assessed as capable of producing exopolysaccharides when the length of mucus strings stretching, as a result of single touching of the isolate colony with a wire-inoculating loop, was 5 mm (or more). The positive results were expressed as “+”, whereas “-” indicated that no mucus (characteristic exopolysaccharide consistency) was visible [18].

#### 2.4.4. Detection of Acid Tolerance in LAB Isolates

The ability of bacteria to survive at low pH was evaluated in triplicate in two types of acidified MRS broths (at pH = 3 and pH = 4) that were inoculated (1% vol/vol) with bacterial cell suspensions. The optical density of the final cell concentration of each LAB isolate (in the exponential growth phase) used for inoculation of the pH-adjusted MRS broths was approximately OD_550_ = 0.125. The tolerance to low pH of the environment was evaluated by measuring OD550 after incubation (24 h at 37 °C in anaerobic conditions). Tested microorganisms with cell suspensions exhibiting OD_550_ of 0.5 (or higher) after incubation were regarded as resistant to low pH.

#### 2.4.5. Hydrophobicity of Bacterial Cells

Hydrophobicity of the cellular surface was determined with the method described by Pérez-Sánchez et al. [19]. After incubation in MRS broth (24 h at 37 °C in anaerobic conditions), the biomass of the analyzed LAB isolates was collected in the stationary phase by centrifugation (6000*× g*/5 min/4 °C). After removing the supernatants, the pellets were washed twice with phosphate buffer (pH 6.5) and suspended (in the same buffer) to obtain all bacterial cell suspensions with optical density (OD_560_) adjusted to 1.0. Subsequently, 3 mL of the suspensions of bacterial cells were transferred into sterile tubes and 1 mL of toluene (Chempur, Piekary Ślaskie, Poland) was added to each sample. Then, the samples were vortexed for 120 s and left at 37 °C for 1 h in order to separate the formed two phases. The aqueous phase was collected and optical density was measured at 560 nm.

Cell hydrophobicity was calculated as the mean percentage decrease in the optical density (OD_560_) of the suspension of the isolates caused by adhesion of bacterial cells to the applied hydrocarbon according to the following formula:

H [%] = [(OD_560_(initial) − OD_560_(with toluene))/(OD_560_(initial)] × 100 (1)

where OD_560_ (initial) and OD_560_ (with toluene) are the absorbances before and after the extraction with toluene, respectively.

#### 2.4.6. Ability to Hydrolyze Bile Salt

The activity of the analyzed bacteria to hydrolyze bile salt was determined using the method described by Gomes et al. [20] with some modifications. The tested isolates were incubated in MRS broth for 12 h and subsequently, bacterial biomasses were collected by centrifugation (8000× *g*/15 min/4 °C) and washed twice with sterile physiological saline. Then, the suspensions of cell cultures (OD_550_ = 0.5) were prepared and spread (10 mL) on the surface of MRS agar containing 0.5% of taurodeoxycholic acid (Sigma-Aldrich, Arklow, Ireland). Afterwards, the MRS agar plates were incubated for 48 h days at 37 °C (in anaerobic conditions). The ability of the isolates to hydrolyze bile salt was detected based on the presence of visible precipitation zones around the colonies [21].

### 2.5. Further Molecular and Biochemical Analysis of Selected LAB Isolates

#### 2.5.1. 16S rRNA Gene Sequencing Analysis

Isolates that were capable of growing at low pH and on a medium with an alternative carbon source exhibiting the ability to produce exopolysaccharides as well as acid and bile salt tolerance were selected for further analysis. This included the identification of tested LAB isolates (to the species level) as well as comparison of the profiles of carbohydrate metabolism by API 50 CH identification system (Biomerieux, Marcy l’Etoile France), which was used according to the manufacturer’s instructions. Moreover, molecular analysis was also performed. Extraction of genomic DNA from pure cultures was performed using Genomic Mini AX Bacteria Spin (A&A Biotechnology, Gdynia, Poland) according to the manufacturer’s protocol. The purity and concentration of the extracted genomic material was determined spectrophotometrically (NanoDrop 2000, Thermo Scientific, Wilmington, DE, USA).

Sequences coding for 16S ribosomal RNA were amplified using the universal primers (27f) 5′-AGAGTTTGATCCTGGCTCAG-3′, and (1495r) 5′-CTACGGCTACCTTGTTACGA-3′ (Genomed S.A., Warsaw, Poland). The PCR reaction was performed according to Gustaw et al. [15]. The PCR reaction mixture (25 μL) contained 100 ng μL of purified chromosomal DNA, 20 pmol of each primer, 12.5 μL of DreamTaq Green PCR Master Mix (2×) (Thermo Scientific, Waltham, MA, USA), and nuclease-free water (adjusted to the final volume).

The amplification was performed in a Labcycler (SensoQuest, Göttingen, Germany) using a program consisting of the following steps: denaturation (4 min/95 °C), 30 cycles including denaturation at 95 °C/30 s, annealing at 48 °C/30 s, and elongation 72 °C/30 s), and final extension (8 min/72 °C) followed by cooling the samples up to 4 °C. The amplification products were electrophoretically separated in 1% agarose gel (Eurx, Gdansk, Poland) containing 0.25% Midori Green DNA Stain (Nippon Genetics Europe, Dueren, Germany). The electrophoresis was conducted in TBE buffer for 1.5 h at 60 V, visualized under UV illumination, and photographed with a digital camera (Gel Doc XR+ Imaging System, BioRad, Waltham, MA, USA).

Amplicons were purified and sequenced with a BigDye Terminator v3.1 Cycle Sequencing Kit (Applied Biosystems, USA) using the capillary 3730xl DNA Analyzer sequencing system (Applied Biosystems, USA). Thereafter, the nucleotide sequences were subjected to further bioinformatic analyses using a DNA Baser Sequence, and BLAST was applied to align the sequences (National Center for Biotechnology Information, Bethesda, MD, USA,). Afterwards, the sequences were compared with sequences available in the GenBank database.

#### 2.5.2. Species-Specific Multiplex-PCR

To distinguish the L. plantarum (Lactiplantibacillus plantarum subsp. plantarum according to new classification), L. pentosus (Lactiplantibacillus pentosus according to new classification), and L. paraplantarum (Lactiplantibacillus pentosus according to new classification) isolates, the Multiplex PCR technique was employed in accordance with Torriani et al. [22] with the use of the specific primers: pREV (5′-TCGGGATTACCAAACATCAC-3′), pentF (5′-CAGTGGCGCGGTTGATATC-3′), paraF (5′-GTCACAGGCATTACGAAAAC-3′), and planF (5′-CCGTTTATGCGGAACACCTA-3′). The oligonucleotides were synthesized by Genomed S.A. (Warsaw, Poland). The PCR products were subjected to electrophoresis, which was conducted in conditions corresponding to those mentioned above.

### 2.6. Statistical Analysis

Statistical analysis was performed using the STATISTICA 13.1 program (StatSoft, Inc., Tulsa, OK, USA), applying Tukey’s HSD test in analysis of variance (ANOVA) to estimate the significance of the differences between the mean values at *p* < 0.05.

## 3. Results

### 3.1. Identification of Autochthonous LAB in Spontaneously Fermented A. bisporus

The detected LAB isolates (Gram-positive, catalase-negative bacteria with an ability to produce lactic acid, bacilli, and cocci with a spherical shape, including bead-like chains) were subjected to initial identification using the MALDI-TOF Biotyper. The identification of the LAB species was based on the alignment of obtained spectra of isolates with the Bruker database (identification over two points within a triple repetition of the experiment was considered highly probable).

The analysis indicated that 58 spectra (which constitutes 52.73% of the isolated LAB) corresponded to *Ln. mesenteroides*, 26.36% to *Lc. lactis*, while 18.18% exhibited high probability to represent *L. plantarum* and only three spectra (that is 2.73% that) were identified as *L. paraplantarum* (Table 1). The results revealed the dominance of *Ln. mesenteroides* in the environment of the spontaneously fermented white button mushrooms.

The isolates exhibited various levels of acid tolerance and a diverse ability to grow on an alternative source of carbon derived from fungal sugars (Table 1). It was noticed that all *Lc. lactis* strains were sensitive to low pH values of the environment, except for E79. Moreover, only four isolates (E64, E68, E74B, E75B) of all the *Lc. lactis* isolates were able to grow on the modified MRS agar (Table 1). The *Ln. mesenteroides* isolate showed a comparably low tolerance to the low value of pH. Furthermore, only E88B and E72C of *Ln. mesenteroides* were able to grow in the medium at pH 3 and pH 4. However, in contrast to *Lc. lactis*, the majority of *Ln. mesenteroides* isolates (52 out of 58) exhibited the ability to grow in the medium containing the fungal material as a substitute of regular glucose. In turn, the *L. plantarum* isolates were characterized by significantly higher tolerance to low pH and only EK2 and E14 were not able to grow at pH = 3. Additionally, 60% of all the *L. plantarum* isolates had an ability to grow on the modified medium (Table 1).

The obtained results of analysis (tolerance to low pH values and growth on modified medium) facilitated the selection of isolates intended for the further stages of the study focused on analysis of their probiotic characteristics and comparison of the acidification properties exhibited by the bacteria during mushroom fermentation.

Comparison of the rate of the decrease in the pH of the fungal material fermented by the selected bacteria isolates indicated that the isolates differed in terms of acidifying properties. The highest acidification dynamics were observed in isolates EK55 and EK4, which, after 24 h of incubation, were able to decrease the pH of the raw material up to 5.06 ± 0.021 and 5.17 ± 0.015, respectively (Table 2). However, none of the microorganisms were able to reduce the pH value (in the experimental conditions) of the fermented material up to pH < 5.

Isolates that were able to growth in MRS broth at pH = 3, pH = 4 as well as on modified MRS agar and exhibited the desirable properties (including the EPS production and bile salt hydrolysis ability presented in Table 2) were subjected to further molecular analysis based on of the analysis the sequence of the conservative gene (see Table S1 in Supplementary Materials).

Most of analyzed sequences of products of amplification 16S rRNA exhibited similarity to *L. plantarum* (isolates EK3, EK5, EK12, E13, EK15, EK51, EK55, E62), while EK4 and EK6 exhibited affinity to bacteria *L. paraplantarum* (see Table S1 in Supplementary Materials).

The results of the multiplex PCR (*rec*A gene) revealed the presence of amplicons with a length of approximately 300 bp (that is characteristic for *L. plantarum*) in eight of ten analyzed bacteria isolates, while 100-bp PCR products were identified only in two samples (isolates EK4 and EK6) that indicate affinity to *L. paraplantarum* (see Figure S1 in Supplementary Materials). The phenotypic test based on carbohydrate fermentation profiles (API 50 CH) classified the tested isolates to *L. plantarum*; however, some differences in the biochemical profiles were observed (see Table S2 in Supplementary Materials). Among the analyzed bacteria, six various patterns of metabolic profiles were noted. The same carbohydrate fermentation profiles showed isolates EK51 and E62. Other isolates that exhibited similar profile were EK55 and EK12. The third group was represented by EK3, EK5, and EK15. In contrast, the remaining isolates (EK4, EK6, and EK13) formed separate profiles (three different patterns of carbohydrate fermentation). It is worth noting that the analysis of 16Sr RNA sequences and the results of the performed multiplex PCR indicated the affinity of EK4 and EK6 isolates to *L. paraplantarum* (that may explain their distinctiveness and considerable differences in profiles of carbohydrate metabolism). Additionally, in the case of EK4, this identification was confirmed by MALDI-TOF Biotyper analysis. 

The results indicate the necessity to perform further, more extensive analyses to determine whether the isolates tested with the same profiles are different strains.

### 3.2. Comparison of the Selected Technological Functional Properties of Selected Autochthonous LAB

For investigation of the selected functional properties, isolated bacteria that exhibited tolerance to low pH values (at pH = 3 and pH = 4) and an ability to grow on modified MRS agar were chosen from all initially identified (MALDI-TOF) isolates. 

The assessment of the ability of the examined microorganisms to hydrolyze bile salts revealed that only five isolates (*L. plantarum* EK11, *Ln. mesenteroides* E24A, *Ln. mesenteroides* E72C, *Ln. mesenteroides* E79B, *Ln. mesenteroides* E88B) did not exhibit such activity (Table 2). In turn, only three (*L. plantarum* EK11, *L. plantarum* EK10, *Ln mesenteroides* E79B) were recognized as EPS non-synthesizing bacteria. Moreover, the analysis of bacterial cell hydrophobicity indicated that all isolates without the capability of exopolysaccharide production and *Ln. mesenteroides* E24A exhibited a hydrophobicity level below 0.1%. In contrast, the highest values of this parameter were noted for *L. plantarum* isolates EK12 (29.59 ± 0.7%), EK55 (28.75 ± 0.551%), and EK5 (27.33 ± 1.516%).

The comparison of the ability to hydrolyze bile salt revealed that not all the *Ln. mesenteroides* isolates demonstrated metabolic activity, ensuring viability in the medium containing 0.5% of bile salts. In contrast, the *L. plantarum* isolates (with the exception of EK11) were characterized by a significantly higher ability to hydrolyze bile salts (Table 2).

## 4. Discussion

### 4.1. Identification and Screening of Autochthonous LAB in Spontaneously Fermented A. bisporus

Greater awareness of the importance of diet and its impact on human health contributes to changes in dietary habits. This significantly increases consumers’ concern with the choice of purchased and consumed food products. It is currently observed that natural and traditional products with a reduced amount of chemical and synthetic substances added at various production stages are desired. For this reason, traditional fermented products (especially plant-based products) are perceived as food with additional desirable pro-health benefits. Moreover, the rising demand for such products is associated with their unique characteristics, which are attributable to traditional production as well as distinctive sensory and organoleptic properties. Furthermore, the application of the starter cultures with probiotic properties rather than regular starter cultures in the food industry may offer additional advantages and may be an interesting approach to enhancement of the use of the industrial process of lactic acid fermentation and production of more health-oriented foods [24,25].

There is ample evidence that the fermentation processes of some food products are carried out by characteristic and innovative starter cultures, which contribute to create the specific and unique characteristics of this type of foodstuff. However, there are no commercial starter cultures (especially with potential health-promoting properties) intended for the fermentation of plant- and mushroom-derived raw materials [25,26,27]. Therefore, this study was focused on the detection and identification of LAB from spontaneously fermented fruiting bodies of white button mushrooms (*A. bisporus*). The selected microorganisms were subjected to preliminary analysis determining some of their functional properties in order identify isolates with the most favorable properties and the highest potential of application as starter cultures (also suitable for lactic acid fermentation of fungal raw material).

The MALDI-TOF identification of the microorganisms indicated the presence of the following LAB species: *Lactococcus lactis, Leuconostoc mesenteroides, Lactiplantibacillus paraplantarum,* and *Lactiplantibacillus plantarum,* with the predominance of *Leuconostoc mesenteroides.* A similar observation was described by other authors, who also noted that the predominance of *Ln. mesenteroides* rapidly declines with the progress of fermentation, due to the sensitivity of these bacteria to low pH acidic conditions [24,28]. Similarly, in analysis of autochthonous functional starter cultures in traditionally produced sauerkraut, Beganović et al. [25] noticed that after three days of fermentation with recorded decline in the pH value, the dominant *Ln. mesenteroides* were replaced by *L. plantarum* and other LAB that completed the process of fermentation. Moreover, it was demonstrated that *Ln. mesenteroides* subsp. *mesenteroides*, belonging to the heterofermentative LAB group, initiates the fermentation of sauerkraut through reducing pH during the production of lactic and acetic acids with a simultaneous reduction in the oxygen level through carbon dioxide production. This bacterium has a considerable influence on the taste, aroma, and quality of fermented cabbage as well [25,29]. This suggests that the prevalence of *Ln. mesentoroides* (noted also in our analysis of spontaneous fermented mushroom material) contributes, to a considerable extent, to the development of organoleptic characteristics of the fermented mushroom-derived products.

The phylogenetic relationships and taxonomic positions of diverse species that possess similar content of GC and exhibit the same nucleotide frequencies can be determined with the use of nucleotide sequences [22,30]. However, it was revealed in the case of some closely related species that the analysis of 16S rRNAs might not be sufficient to provide information on recent evolutional changes (mutations), since they are too recent to be fixed in such slowly diverging sequences [22]. As suggested in scientific reports, the amplification and sequencing of the 16S rRNA gene is often not sufficient to identify and distinguish *L. plantarum, L. paraplantarum*, and *L. pentosus*. Therefore, the species-specific multiplex-PCR proposed by Torriani et al. [22] was implemented in our investigations (since *rec*A can tolerate mutations that either do not or only slightly alter its products). The results of the analysis proved that all the multiplex PCR products were specific to *L. plantarum* species, except EK4 and EK6 that exhibited affinity to *L. paraplantarum*.

### 4.2. Comparison of the Selected Technological and Functional Properties of the Chosen Autochthonous LAB

The results of the preliminary analysis (ability of the isolates to grow on medium with an alternative carbon source containing mushroom-derived material and the dynamics of acidification of the mushroom material during fermentation) suggest that some of the tested endogenous isolates of LAB (especially *L. plantarum* EK55 and *L. paraplantarum* EK4) can potentially be applied for the formulation of starter cultures (monoculture or component of multicultural composition) suitable for the lactic acid fermentation of plant or fungal raw material.

One of the main parameters determining the properties of fermented food is the pH value, which not only influences the flavor but also has an effect on the microbiological stability against pathogens and prevents food quality deterioration [31]. *L. plantarum* is perceived as the most acid tolerant species predominant in sauerkraut fermentation, especially in the late homofermentative phase of this process [32,33]. However, it was noticed that none of the tested autochthonous bacterial isolates from white button mushrooms were able to reduce the pH of the fermented product below 5 after 24 h at 37 °C. This might be a consequence of the insufficient time of incubation (too short a fermentation process) or an inadequate dose of inoculum for the fermentation of this type of raw material. Khaskheli et al. [34] noted that pH value of *Auricularia auricular* fermented at 26 ± 4 °C for about 3 to 4 weeks reached from 4.5 to 5.5. Probably, a longer time of fermentation would have resulted in pH < 5 of the fermented fruiting bodies of white button mushrooms (*A. bisporus*).

The selected isolates were analyzed in order to evaluate the desirable features (including resistance to bile salt, acidic tolerance, hydrophobicity) in LAB probiotics. The investigations revealed that 11 of the 16 isolates were able to grow on MRS agar containing 0.5% of bile salts. It is worth mentioning that supplementation of the medium with bile salts up to 0.3% is deemed to be critical and sufficiently high to screen for resistant strains [35,36]. The results indicate that *Ln. mesenteroides* (E24A, E72C, E79B, E88B) and *L. plantarum* EK11 were sensitive to analyzed bile salts concentration.

It has been suggested that bacterial cell hydrophobicity is influenced by the ability to synthesize exopolysaccharides [37]. It is also claimed that extracellular proteins in EPSs have major relevance in the protective function, as they contribute to the bacterial surface hydrophobicity [38]. The results of the estimation of the ability of the tested isolates to produce exopolysaccharides suggested that EPS non-producing bacteria were also characterized by very low cell hydrophobicity (below 0.1%). This is in agreement with results described by Xia et al. [38], who analyzed extracellular polymeric substances protecting *Escherichia coli* from organic solvents and found that an increase in hydrophobicity was correlated with differences in EPS content.

The results obtained in our study suggest that especially *L. plantarum* (EK5, EK12, EK55) and *L. paraplantarum* EK4 can be further analyzed to determine their potential applications in the fermented food industry as starter cultures. Moreover, in this preliminary study, these microorganisms exhibited survivability at low pH, high hydrophobicity of the bacterial cell, ability to produce EPS, and ability to hydrolyze bile salt, which represent part of the desired probiotic properties. Therefore, the obtained findings of this preliminary study have particular importance especially in regards to the possibility of the application of a biological preservation of mushroom fruiting bodies by lactic acid fermentation using determined LAB isolates, which currently is not popular or developed on an industrial scale [5].

The application of selected LAB isolates provides a proper and repeatable fermentation process. Additionally, lactic fermentation is expected to improve the health values of the mushrooms at the same time through the presence of viable lactic acid bacteria. The promising results of this preliminary research prompt further extensive analyses of the identified autochthonous LAB isolated from the spontaneously fermented fruiting bodies of white button mushrooms. Therefore, the technological and functional features exhibited by isolated LAB have to be further studied in the food system (in various food matrixes, including mushrooms- and plant-derived raw materials or even milk). It should be also verified whether these are different strains and, if so, the level of their diversity should be assessed. Additionally, their properties as a probiotic starter culture should be determined in terms of their application in the production of plant- and mushroom-based functional foodstuff.

Moreover, due to the fact that nowadays, the application of technologically functionalized starter cultures is more desirable (because of the greater survivability and viability of the microorganisms in environmental conditions) [39,40], developing a proper form of starter cultures, allowing the maintenance of appropriate levels of bacteria activity in the material subjected to the fermentation process, is a great challenge and subject of prospective study.

## 5. Conclusions

The findings of the initial study exhibit that spontaneously fermented *A. bisporus* fruiting bodies may be an alternative source of new LAB isolates exhibiting adaptation to the acidic environment and desirable functional properties that may contribute to development of potentially probiotic starter cultures. Among autochthonous LAB (isolated from the tested mushroom raw material), *Ln. mesenteroides* predominated in spontaneously fermented *A. bisporus*, while *L. paraplantarum, L. plantarum*, and *Lc. lactis* were less abundant. Considering the parameters analyzed in the research, isolates EK55 and EK4 exhibited desired features as well as functional and technological potential; therefore, they are intended for further research analyzing these bacteria’s suitability in the formulation of novel starter cultures. However, there is still a need to continue the study via a wide range of interdisciplinary analyses for full characterization of the isolates at the strain level (unambiguously differentiating the strains with exhibited by them desired features) and verify their technological and functional properties determining their further applications.

## Figures and Tables

**Table 1:** Species identification (MALDI-TOF Biotyper) and comparison of the characteristics of lactic acid bacteria (LAB).

Isolate ID *	Morphological Form (Shape)	Gram Stain	Lactic Acid Production	Catalase Reaction	Species ** Identified by MALDI-TOF	Growth in		
						MRS Broth at pH = 3	MRS Broth at pH = 4	MSodified MRS Agar
EK1, E56, E57, E58, E59, E60, E61	R	P	+	-	*Lactiplantibacillus plantarum*	+	+	_
EK2, E14	R	P	+	-	*Lactiplantibacillus plantarum*	-	+	+
EK3, EK5, EK6, EK10, EK11, EK12, EK13, EK15, EK51, EK55, E62	R	P	+	-	*Lactiplantibacillus plantarum*	+	+	+
EK7, EK8, EK9, E21, E22, E26, E65, E66, E67, E100, E101, E102, E103, E104, E120, E121, E122, E123, E124, E73B, E125, E126, E127, E128	C	P	+	-	*Lactococcus lactis*	-	-	-
E64, E68, E74B, E75B	C	P	+	-	*Lactococcus lactis*	-	-	+
E79A	C	P	+	-	*Lactococcus lactis*	+	+	-
E23, E24A, E25, E96C, E76B	C	P	+	-	*Leuconostoc mesenteroides*	-	-	-
E88B, E72C	C	P	+	-	*Leuconostoc mesenteroides*	+	+	+
E71C, E76C, E79C, E80C, E81B, E82B, E83B, E84A, E96C, E71A, E73A, E72A, E74A, E75A, E76A, E78A, E71B, E84B, E93C, E94C, E95C, E81A, E83A, E82A, E80A, E86B, E73C, E78B, E77B, E77A, E79B, E80B, E89B, E74C, E75C, E78C, E81C, E82C, E83C, E84C, E72B, E85C, E86C, E87C, E88C, E89C, E90C, E91C, E92C, E87B, E85B	C	P	+	-	*Leuconostoc mesenteroides*	-	-	+
E53, E54	R	P	+	-	*Lactiplantibacillus paraplantarum*	-	-	+
EK4	R	P	+	-	*Lactiplantibacillus paraplantarum*	+	+	+

Explanatory notes: * Isolate name; ** according to the classification [23], *Lactobacillus plantarum* has been renamed to *Lactiplantibacillus plantarum subsp*. *plantarum*; R—rod; C—cocci spherical shape (bead-like chains); P—positive results of Gram staining; “+” means that the isolate exhibited the ability; “-“ means lack of the ability.

**Table 2:** Comparison of selected functional properties.

Isolate	pH of Mushroom Material Fermented by the Isolate			Ability to EPS Production	Ability to Hydrolyze Bile Salt	Cell Hydrophobicity [%]
	After 3 h	After 7 h	After 24 h			
EK3	6.27 ± 0.07 ^abB^	6.33 ± 0.012 ^efB^	6.02 ± 0.026 ^jA^	+	+	0.34 ± 0.292 ^a^
EK4	6.36 ± 0.052 ^cdefB^	6.35 ± 0.010 ^fgB^	5.17 ± 0.015 ^bA^	+	+	25.34 ± 0.407 ^e^
EK5	6.37 ± 0.025 ^cdefC^	6.29 ± 0.010 ^bcdB^	6.20 ± 0.015 ^kA^	+	+	27.33 ± 1.516 ^f^
EK6	6.30 ± 0.021 ^abcdA^	6.39 ± 0.006 ^hA^	5.70 ± 0.010 ^eA^	+	+	0.37 ± 0.112 ^a^
EK10	6.42 ± 0.053 ^aC^	6.23 ± 0.006 ^cdeB^	5.69 ± 0.010 ^gA^	-	+	n.d.
EK11	6.54 ± 0.089 ^abcB^	6.19 ± 0.006 ^bcdB^	5.71 ± 0.010 ^hA^	-	-	n.d.
EK12	6.39 ± 0.055 ^efB^	6.34 ± 0.010 ^efgB^	5.40 ± 0.015 ^cA^	+	+	29.59 ± 0.700 ^g^
EK13	6.39 ± 0.026 ^defB^	6.37 ± 0.010 ^ghB^	6.01 ± 0.021 ^jA^	+	+	0.11 ± 0.113 ^a^
EK15	6.48 ± 0.071 ^bcdeC^	6.21 ± 0.006 ^abB^	5.70 ± 0.010 ^fA^	+	+	4.66 ± 0.918 ^b^
EK51	6.34 ± 0.006 ^bcdefC^	6.28 ± 0.010 ^abcB^	5.79 ± 0.010 ^fA^	+	+	8.83 ± 0.104 ^c^
EK55	6.35 ± 0.006 ^bcdefB^	6.32 ± 0.015 ^efB^	5.06 ± 0.021 ^aA^	+	+	28.75 ± 0.551 ^fg^
E62	6.31 ± 0.012 ^abcdA^	6.29 ± 0.006 ^bcB^	5.94 ± 0.010 ^iA^	+	+	1.52 ± 0.435 ^a^
E24A	6.34 ± 0.017 ^bcdeB^	6.27 ± 0.015 ^bcdB^	5.77 ± 0.006 ^fgA^	+	-	n.d.
E72C	6.34 ± 0.006 ^bcdeB^	6.32 ± 0.010 ^defB^	5.54 ± 0.010 ^dA^	+	-	16.63 ± 2.217 ^d^
E79B	6.29 ± 0.017 ^abcB^	6.27 ± 0.006 ^abB^	5.67 ± 0.010 ^eA^	-	-	n.d.
E88B	6.35 ± 0.035 ^bcdefC^	6.25 ± 0.006 ^aB^	5.68 ± 0.010 ^eA^	+	-	14.68 ± 0.151 ^d^
Control	6.43 ± 0.017 ^fB^	6.42 ± 0.010 ^iAB^	6.4 ± 0.010 ^lA^			

Explanation notes: The results are given as mean values ± standard deviation (x ± s/SD; n = 5). The following uppercase letters (A–C) express significant differences (*p* < 0.05) in the same row, while the lowercase letters (a–l) express significant differences (*p* < 0.05) between values in the same column; n.d.—not detected (value below 0.1%).

## Supplementary Materials

The following are available online at https://www.mdpi.com/2304-8158/9/11/1631/s1. Table S1: The results of comparison of the obtained 16S rRNA sequences of analyzed isolates with sequences available in the GenBank database.; Table S2: The comparison of the profiles of carbohydrate fermentation exhibited by the tested isolates.; Figure S1: Electrophoretic separation the *products of amplification rec*A gene (line M1 and M2—molecular markers expressing mass in bp; *line 1*—negative control sample; *line 2—*EK3; *line 3*–EK4; *line 4—*EK5; *line 5—*E*K*12; *line 6*—E*K*15; *line 7*—EK13*; line 8–EK51; line 9—EK6; line 10*—EK55; line*11—*E62).
