# Supplementary material for: Spontaneously Fermented Fruiting Bodies of Agaricus bisporus as a Valuable Source of New Isolates of Lactic Acid Bacteria with Functional Potential
Source: Foods. 2020 Nov 8;9(11):1631. doi: 10.3390/foods9111631 (PMC7695194; doi:10.3390/foods9111631)
Supplement: Supplementary file 1 [file foods-09-01631-s001.pdf]

Table S1. The results of comparison the obtained 16S rRNA sequences of analysed isolates with sequences available in the GenBank database.

The Analysed Sequence of 16S rRNA of Isolate	Sequences Producing Significant Alignments	Identities [%]	Accession
EK3	*L. plantarum strain* TMW 1.1308 chromosome, complete genome *Lactobacillus plantarum* strain SRCM101511 chromosome, complete genome	99.9	MT457699.1
EK4	*L. paraplantarum* strain 2215 16S ribosomal RNA gene, partial sequence	100	MT604711.1
EK5	*L. plantarum* strain 1971 16S ribosomal RNA gene, partial sequence	94.99	MT597777.1
EK6	*L. paraplantarum* strain shebah-401 16S ribosomal RNA gene, partial sequence	100	MN602521.1
EK12	*L plantarum* strain M4D12 16S ribosomal RNA gene, partial sequence	92.90	MH714779.1
EK13	*L. plantarum* strain 7232 16S ribosomal RNA gene, partial sequence	99.86	MT645511.1
EK15	*L. plantarum* strain 2975 16S ribosomal RNA gene, partial sequence	99.71	MT611908.1
EK51	*L. plantarum* strain MLG6-31 16S ribosomal RNA gene, partial sequence	99.29	MT473428.1
EK55	*L. plantarum* strain MG5289 16S ribosomal RNA gene, partial sequence	99.23	MN368072.1
E62	*L. plantarum* strain FFNL190 16S ribosomal RNA gene, partial sequence	99.03	MT109194.1

Table S2. The comparison the profiles of carbohydrate fermentation exhibited by the tested isolates.

Carbohydrate (Source of Carbon in Medium)	The Analyzed LAB Isolate									
	EK51	EK55	E62	EK3	EK4	EK5	EK6	EK12	EK13	EK15
Control	-	-	-	-	-	-	-	-	-	-
Glycerol	-	-	-	-	-	-	-	-	-	-
Erythritol	-	-	-	-	-	-	-	-	-	-
D-Arabinose	-	-	-	-	-	-	-	-	-	-
L-Arabinose	-	-	-	-	-	-	-	-	-	-
Ribose	+	+	+	+	+	+	+	+	+	+
D-Xylose	-	-	-	-	-	-	-	-	-	-
L-Xylose	-	-	-	-	-	-	-	-	-	-
Adonitol	-	-	-	-	-	-	-	-	-	-
β-Methyl-xyloside	-	-	-	-	-	-	-	-	-	-
Galactose	+	+	+	+	+	+	+	+	+	+
D-Glucose	+	+	+	+	+	+	+	+	+	+
D-Fructose	+	+	+	+	+	+	+	+	+	+
D-Mannose	+	+	+	+	+	+	+	+	+	+
L-Sorbose	-	-	-	-	-	-	-	-	-	-
Rhamnose	-	-	-	-	+	-	+	-	-	-

Dulcitol	-	-	-	-	-	-	-	-	-	-
Inositol	-	-	-	-	-	-	-	-	-	-
Mannitol	+	+	+	+	+	+	+	+	+	+
Sorbitol	+	+	+	+	+	+	+	+	+	+
α-Methyl-D-mannoside	+	+	+	+	+	+	+	+	+	+
α-Methyl-D-glucoside	-	-	-	-	-	-	-	-	-	-
N-acetyl glucosamine	+	+	+	+	+	+	+	+	+	+
Amygdaline	+	+	+	+	+	+	+	+	+	+
Arbutine	+	+	+	+	+	+	+	+	+	+
Esculine	+	+	+	+	+	+	+	+	+	+
Salicine	+	+	+	+	+	+	+	+	+	+
Cellobiose	+	+	+	+	+	+	+	+	+	+
Maltose	+	+	+	+	+	+	+	+	+	+
Lactose	+	+	+	+	+	+	+	+	+	+
Melibiose	+	+	+	+	+	+	+	+	+	+
Saccharose	+	+	+	+	+	+	+	+	+	+
Trehalose	+	+	+	+	+	+	+	+	+	+
Inuline	-	+	-	+	-	+	+	+	-	+
Melezitose	+	+	+	+	+	+	+	+	+	+
D-Raffinose	+	+	+	-	+	-	+	+	+	-
Starch	-	-	-	-	-	-	-	-	-	-
Glycogene	-	-	-	-	-	-	-	-	-	-
Xylitol	-	-	-	-	-	-	-	-	-	-
β-Gentiobiose	+	+	+	+	+	+	+	+	+	+
D-Turanose	-	-	-	-	-	-	-	-	-	-
D-Lyxose	-	-	-	-	-	-	-	-	-	-
D-Tagatose	-	-	-	-	-	-	-	-	-	-
D-Fucose	-	-	-	-	-	-	-	-	-	-
L-Fucose	-	-	-	-	-	-	-	-	-	-
D-Arabitol	-	+	-	+	+	+	+	+	+	+
L-Arabitol	-	-	-	-	-	-	-	-	-	-
Gluconate	+	+	+	+	+	+	+	+	+	+
2-ceto-gluconate	-	-	-	-	-	-	-	-	-	-
5-ceto-gluconate	-	-	-	-	-	-	-	-	-	-

Explanation notes: „+" positive results; „-" negative results (no changes have been observed)
